# Stress-Induced Activation of Heterochromatic Transcription

**DOI:** 10.1371/journal.pgen.1001175

**Published:** 2010-10-28

**Authors:** Mireille Tittel-Elmer, Etienne Bucher, Larissa Broger, Olivier Mathieu, Jerzy Paszkowski, Isabelle Vaillant

**Affiliations:** 1 Department of Plant Biology, University of Geneva, Geneva, Switzerland; 2 Centre National de la Recherche Scientifique (CNRS), UMR 6247 - GReD - INSERM U 931, Clermont Université, Aubière, France; Stanford University, United States of America

## Abstract

Constitutive heterochromatin comprising the centromeric and telomeric parts of chromosomes includes DNA marked by high levels of methylation associated with histones modified by repressive marks. These epigenetic modifications silence transcription and ensure stable inheritance of this inert state. Although environmental cues can alter epigenetic marks and lead to modulation of the transcription of genes located in euchromatic parts of the chromosomes, there is no evidence that external stimuli can globally destabilize silencing of constitutive heterochromatin. We have found that heterochromatin-associated silencing in *Arabidopsis* plants subjected to a particular temperature regime is released in a genome-wide manner. This occurs without alteration of repressive epigenetic modifications and does not involve common epigenetic mechanisms. Such induced release of silencing is mostly transient, and rapid restoration of the silent state occurs without the involvement of factors known to be required for silencing initiation. Thus, our results reveal new regulatory aspects of transcriptional repression in constitutive heterochromatin and open up possibilities to identify the molecular mechanisms involved.

## Author Summary

In eukaryotic cells, DNA is packaged into chromatin that is present in two different forms named euchromatin and heterochromatin. Gene-rich euchromatin is relaxed and permissive to transcription compared with heterochromatin that essentially contains transcriptionally inert non-coding repeated DNA. The silent state associated with heterochromatin correlates with the presence of distinctive repressive epigenetic modifications. Mutations in genes required for maintenance of these epigenetic marks reactivate heterochromatin transcription, which is otherwise maintained silent in a highly stable manner. In this paper, we defined a specific temperature stress that leads to genome-wide transcriptional activation of sequences located within heterochromatin of *Arabidopsis thaliana*. Unexpectedly, release of silencing occurs in spite of conservation of the repressive epigenetic marks and independently of common epigenetic regulators. In addition, we provide evidence that stress-induced transcriptional activation is mostly transient, and silencing is rapidly restored upon return to optimal growth conditions. These results are important in that they disclose the dynamics of silencing associated with heterochromatin as well as the existence of a new level of transcriptional control that might play a role in plant acclimation to changing environmental conditions.

## Introduction

Chromatin can adopt conformations that were first defined cytologically as condensed heterochromatin and open euchromatin [1]. Subsequent genomic studies characterized euchromatin as gene rich and transcriptionally active, and heterochromatin as inert matter, mostly holding transcriptionally silent repeats, remnants of transposons and DNA sequences without clearly defined functions. It is, however, remarkable that a very large proportion of genomic DNA is packaged into heterochromatin, often overwhelming the amounts of DNA associated with euchromatin. Such disproportion is especially apparent for large mammalian genomes [2]–[4] and those of plants, where in maize, for example, approximately 85% of DNA resides in heterochromatin [5], [6]. It is inherently difficult to assign sequence-specific activities and functions to heterochromatic DNA due to the high degree of repetitiveness, which may even prevent unequivocal assembly of sequences at these parts of chromosomes.

Nevertheless, the oldest and best-documented functions of heterochromatin relate to basic chromosomal activities such as the formation and behavior of centromeres and telomeres [7]–[9]. It has also been postulated that suppressive properties of heterochromatin towards transcription are essential for silencing of transposons, which are inactivated when inserted into heterochromatic DNA and passively transmitted through mitosis and meiosis thus harmless to the host genome [10]. The maintenance of compact and inert heterochromatin seems to be correlated with the propagation of particular covalent modifications of DNA and histones. These modifications, termed epigenetic marks, are propagated together with replicating DNA. In plants and mammals, heterochromatic DNA is densely methylated at cytosine residues (^m^C) and is associated with deacetylated histones H3 methylated at lysine 9 (H3K9me). In euchromatin, DNA methylation levels are lower and H3 gains acetylation and methylation at lysine 4 (H3K4me) losing H3K9me [11], [12].

Transcriptional responses to a plethora of environmental stimuli have been documented for many euchromatin-associated genes and/or gene networks. These responses seem to be specific to particular environmental challenges. Since only a subset of genes undergoes activation or suppression in response to a given challenge, this provides an expression fingerprint that allows for rapid adaptation to a unique or combinations of environmental stress [13]–[18]. These responses have been associated with alterations in epigenetic regulatory mechanisms, such as changes in the distribution of DNA methylation, histone modifications [19] or populations of regulatory small RNAs [20], [21]. The involvement of small RNAs (siRNAs and miRNAs) leading to modifications of epigenetic marks at target genes and/or degradation of mRNAs or the translational inhibition by post-transcriptional gene silencing (PTGS) seem to play important roles in stress responses [22]. One of the best-studied examples is an arms race occurring during viral infection, where plants and invertebrates deploy RNA silencing for their defense, which involves the production of virus-derived small interfering RNAs (viRNAs) [23]. In plants, siRNAs (including natural antisense transcripts-derived siRNAs, nat-siRNAs) and miRNAs have been shown to participate in antibacterial defense (reviewed in [24], [25]), in abiotic stress responses, and in reactions to nutrient deprivation (reviewed in [22], [24]). Notably, these adaptations seem to occur in a transient fashion with kinetics similar to the regulation of transcription by transcription factors. Therefore, it has been difficult to define whether epigenetic mechanisms associated with transcriptional gene responses are causal or secondary to gene activation. Nevertheless, although stress-induced alterations in euchromatic gene transcription are well documented, there is only limited evidence so far that environmental stimuli can alleviate the profound suppression of transcription in heterochromatin [26], which seems to be constitutively silenced by multilayer of epigenetic control. Although this secures transcriptional gene silencing (TGS) in these chromosomal regions, transcriptional activity at loci residing in heterochromatin is regained in a number of mutants affecting epigenetic regulation (see for instance [27]–[31]). Moreover, results with combinations of multiple mutations in genes involved in epigenetic regulation illustrate the very complex strategy securing stability, robustness and, therefore, persistence of transcriptional suppression in heterochromatin [29], [30], [32]–[34]. The reasons for such tight transcriptional suppression are not clear, but it can be envisaged that prevention of transcription in heterochromatin is required for the structural stability and the function of centromeric, pericentromeric and telomeric regions. In addition, transposon-derived transcription should ideally remain suppressed to prevent their mobility. However, analyses of *Arabidopsis* mutants with distorted heterochromatin structure and released transcriptional suppression in heterochromatin do not fully support these hypotheses. Two mutations that most drastically affect heterochromatin structure and its transcriptional silencing, *met1* and *ddm1,* do not evoke chromosome losses or instantaneous transposon movement despite their transcriptional activation [35]–[40]. *MET1* encodes maintenance DNA methyltransferase and *DDM1* a chromatin remodeling ATPase [41]–[43]. Both MET1 and DDM1 are required for propagation of DNA methylation at cytosines in CG sequences (^m^CG) [41]–[43], which seems to be the most stable epigenetic mark essential for transgenerational epigenetic inheritance in *Arabidopsis* [32]. Interestingly, although centromeric heterochromatin in both these mutants is decondensed and transcriptionally active, no obvious deficiencies in the functions of centromeres or telomeres have been reported. It has also been shown that transcriptional activation of transposons is not directly related to their movement, which seems to be controlled also at the posttranscriptional level [35], [36]. Therefore, it remains largely unclear why heterochromatin structure and transcriptional silencing are so firmly maintained and, as a consequence, it is also unclear whether this part of the genome is at all able to either perceive or respond at the transcriptional level to environmental stimuli.

Here we describe an experimental system designed to test the influence of various environmental challenges on transcriptional suppression in *Arabidopsis* heterochromatin. The system exploits the well-documented observation that multicopy transgenic inserts tend to acquire properties and epigenetic marks characteristic of constitutive heterochromatin. Such silent transgenic loci can be activated in mutants affecting epigenetic regulation of endogenous targets residing in heterochromatin. We applied a series of abiotic stresses to transgenic *Arabidopsis* plants and used the activation of an originally silent transgenic locus as readout for the destabilization of heterochromatic TGS. This approach allowed the definition of environmental stress conditions that not only destabilize transgene silencing but also result in genome-wide reactivation of endogenous heterochromatic loci. However, silencing release was mostly transient and was rapidly restored upon return to normal growth conditions. This transient activation of heterochromatic transcription occurred genome-wide and was not associated with changes in DNA methylation or repressive histone modifications that were examined at a subset of reactivated loci. Intriguingly, mutations in common epigenetic gene silencing regulators, including those involved in *de novo* DNA methylation or H3K9me, did not prevent rapid resilencing after stress treatments.

## Results

### Selection of abiotic stress conditions releasing transcriptional gene silencing

In order to define stress conditions able to release TGS, we used the well-characterized transgenic line L5 of *Arabidopsis*, which contains a single locus consisting of 3–4 copies of a methylated and silenced marker gene encoding β-glucuronidase (GUS) linked to the 35S promoter of the Cauliflower Mosaic Virus [44], [45]. Silencing of the *GUS* transgene is released in mutants deficient for TGS maintenance [44]–[48].

L5 plants were exposed at differed developmental stages to salt, osmotic and temperature stresses of gradually increasing severity and TGS release was monitored at the transgenic *GUS* locus using histochemical GUS assays. Treatments provoking salt or osmotic stress had no influence on the stability of TGS even close to the LD50 (not shown). In contrast, thermal stress led to destabilization of silencing at the GUS locus, similar to a recent study using different stress conditions [26]. The degree of silencing release was related to a particular combination of temperature shifts. The experiments delineating the most effective thermal stress conditions for TGS release are described below.

**Figure 1:**
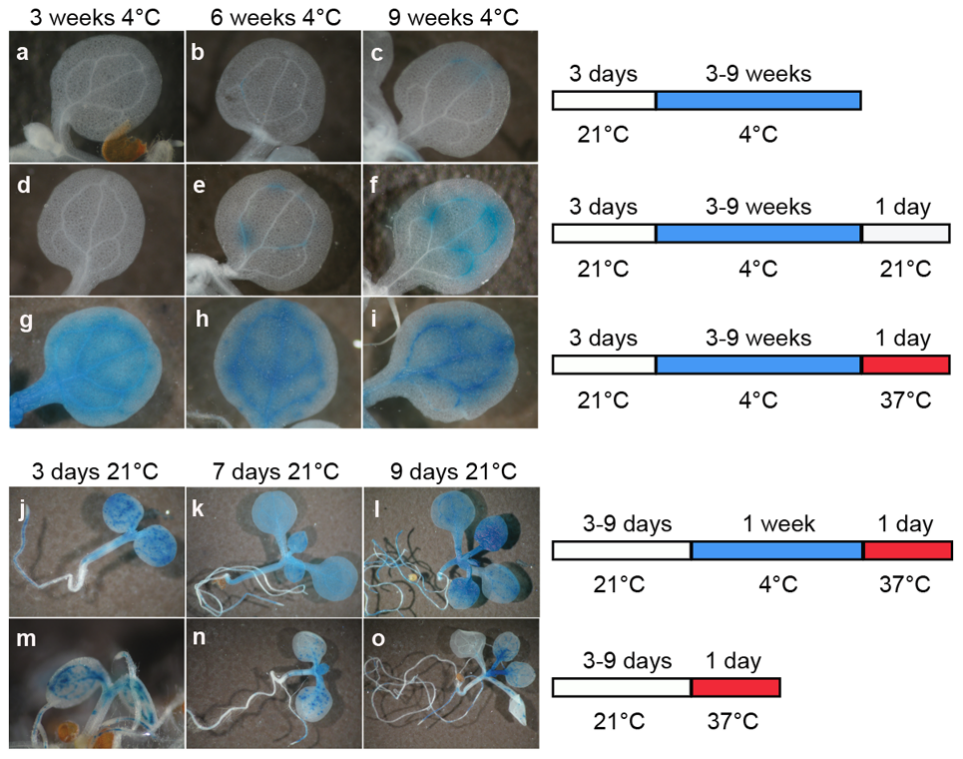
A temperature shift can release transcriptional silencing of a transgenic locus. Representative images of histochemical staining for GUS activity (left) performed on seedlings grown under the conditions defined on the right. Plants grown for 3 days at 21°C were transferred to 4°C for 3–9 weeks (a–c) and then shifted to either 21°C (d–f) or 37°C (g–i) for 1 day. Seedlings at 3, 7, and 9 days post-sowing were transferred at 4°C for 1 week and shifted to 37°C for 1 day (j–l), or directly shifted to 37°C for 1 day omitting the cold treatment (m–o).

**Figure 2:**
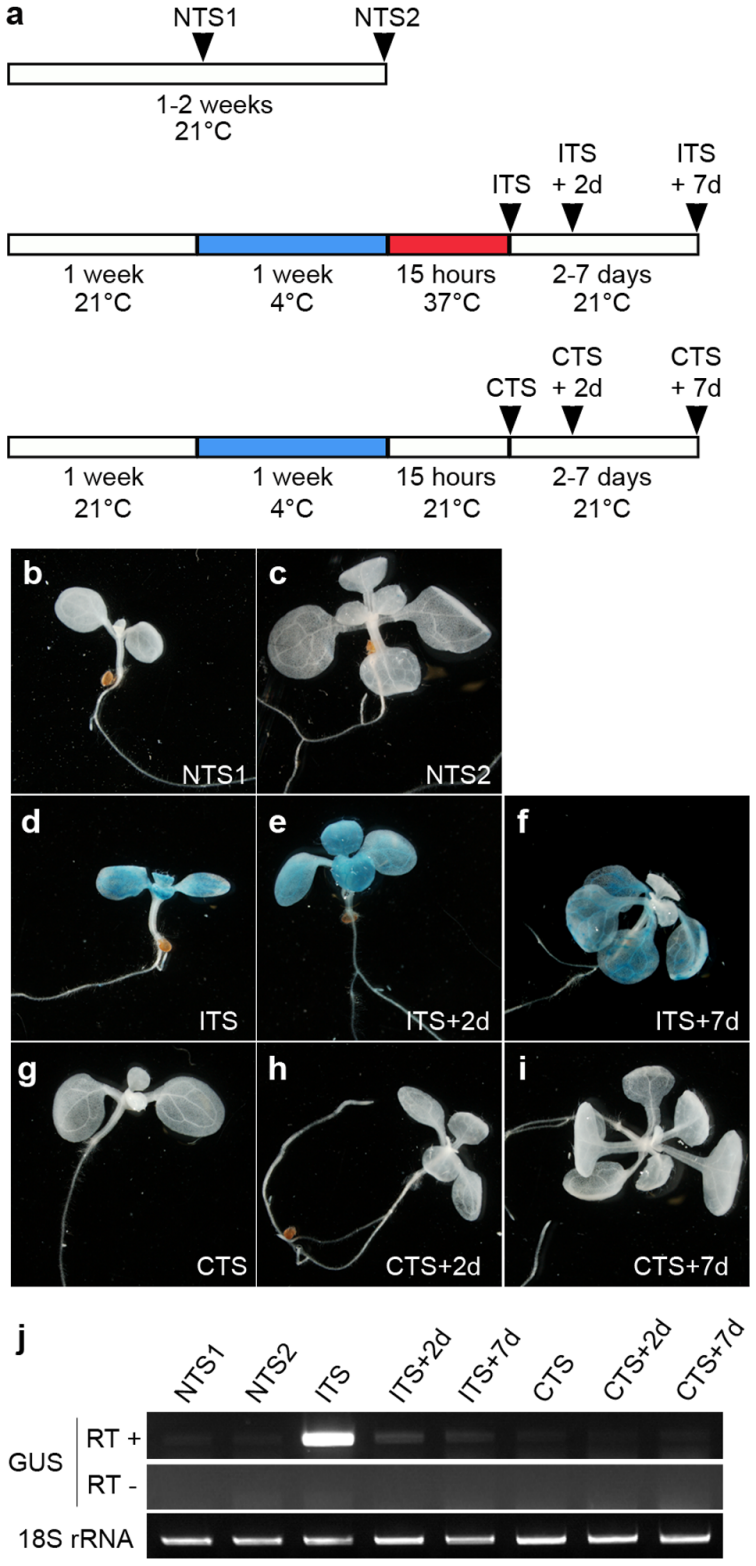
The ITS-induced release of transcriptional silencing is transient. (a) Experimental scheme of the control and stress treatments. (b–i) Representative images of histochemical staining for GUS activity performed on seedlings grown under the indicated conditions. (j) Reverse-transcription-PCR detection of GUS transcripts from total RNA of the indicated samples. Amplification of 18S rRNA was used to normalize the amounts of RNA template. Negative controls lacked reverse transcriptase (RT -).

Three-day-old seedlings were exposed to a long cold period (4°C) known to alter DNA methylation [49] and also to influence silencing mediated by polycomb-group proteins, which is best illustrated by the vernalization process [50]–[52]. Cold-exposed and control seedlings were subsequently subjected to histochemical GUS staining (Figure 1A–1C). Three or 6 weeks of cold treatment did not destabilize GUS silencing (Figure 1A and 1B); however, seedlings transferred to 4°C for 9 weeks showed weak TGS release manifested by occasional patches of GUS staining in a proportion of seedlings (Figure 1C). When seedlings were returned to 21°C for 24 h following the cold treatment, GUS staining was also detected in seedlings placed in the cold for only 6 weeks, and this shift led to increased GUS staining intensity in plants grown at 4°C for 9 weeks (Figure 1D–1F). Therefore, we concluded that, in addition to cold treatment, a temperature shift may also contribute to the release of TGS. To test this, we extended the range of the temperature shifts from 21°C to 37°C (Figure 1G–1I). While no GUS expression was observed in plants kept in the cold for only 3 weeks and then placed at 21°C for 24 h, a temperature shift to 37°C instead of 21°C resulted in very clear GUS activity (Figure 1G). This activity remained at a similar level when longer cold periods were applied, suggesting that the length of the cold period preceding the temperature shift to 37°C was not a limiting factor for the release of TGS (Figure 1H and 1I). To further examine this, we shortened the cold period to 1 week or even omitted it prior to the temperature shift to 37°C. For these experiments, we used seedlings at three stages (3, 7, and 9 days after sowing) in order to assess also whether silencing release can be effective over a broader span of early plant development. One week of cold treatment followed by a shift to 37°C for 24 h was sufficient to release silencing of *GUS* locus at all three developmental stages of the seedlings (Figure 1J–1L). Omission of the cold period prior to the shift to 37°C resulted not only in less uniform and less pronounced TGS release (Figure 1M–1O) but also caused plant lethality (not shown). Therefore, the cold period before the shift to high temperature increased both plant viability and the amplitude of TGS suppression. Shortening the period at 37°C to 15 h permitted most of this treatment (12 h) to be performed during the light phase of the applied photoperiod and promoted plant survival. The shortening of the time at 37°C had no influence on the degree of silencing release (Figure 1K and Figure 2D and data not shown).

For all subsequent experiments, a standardized treatment was used in which 1-week-old seedlings grown at 21°C were transferred to 4°C for 1 additional week and subsequently subjected to the shift to 37°C for 15 h (‘Inductive Temperature Shift’ or ITS). Cold-treated plants exposed to a shift to 21°C for 15 h were used as controls (‘Control Temperature Shift’ or CTS). Since plants placed at 4°C stopped growing almost completely, we therefore used two ‘No Temperature Shift’ (NTS) controls in which plants were harvested 1 week after sowing (NTS1) or 2 weeks after sowing (NTS2), i.e. after growing at 21°C for the same period as test plants subjected to the temperature shifts. The plants subjected to temperature shifts were harvested at three time points: directly after treatment at 37°C or control treatment at 21°C or after 48 h (2 days) or 7 days following the treatments, to allow recovery during further growth at 21°C. The experimental schemes are shown in Figure 2A.

### Stress releases TGS only transiently

Release of silencing at the *GUS* locus occurred neither in control plants without a temperature shift (NTS1 NTS2; Figure 2B and 2C) nor in plants moved from 4°C to 21°C (CTS; Figure 2G). In contrast, the silent *GUS* locus became active in plants moved from 4°C to 37°C (ITS; Figure 2D). Therefore, only the ITS treatment was able to release *GUS* silencing as revealed by histochemical staining. The CTS and ITS treated plants were at the same developmental stage, when the first pair of true leaves were emerging, and were treated in parallel. Therefore, factors other than ITS itself that may have contributed to the TGS release, such as specific developmental stress responses, can be excluded. Following ITS, GUS activity was still detected in plants grown for an additional 48 h (ITS+2d) and even for 7 days (ITS+7d) (Figure 2E and 2F). In contrast to a recent study showing development of GUS-positive new leaves 1 week after a heat stress of 48 h at 42°C [26], we found that new leaves developed in ITS+7d plants had no GUS activity. This suggests that the transgene was resilenced relatively rapidly and that the persistence of GUS activity in cotyledons and leaves subjected to the ITS results from residual GUS activity retained in these tissues. Indeed, β-glucuronidase has been shown previously to be a rather stable protein [53]. Additional RT-PCR analysis of *GUS* transcripts further supported very rapid resilencing of the locus. Indeed, *GUS* transcripts, which accumulated directly after the ITS, were already almost undetectable 2 days after the ITS (Figure 2J). Importantly, these transcripts were absent in CTS plants, which further confirms that elevation of temperature to 37°C was critical for destabilization of transcriptional gene silencing at this locus.

### ITS releases transcriptional silencing of endogenous chromosomal targets

**Figure 3:**
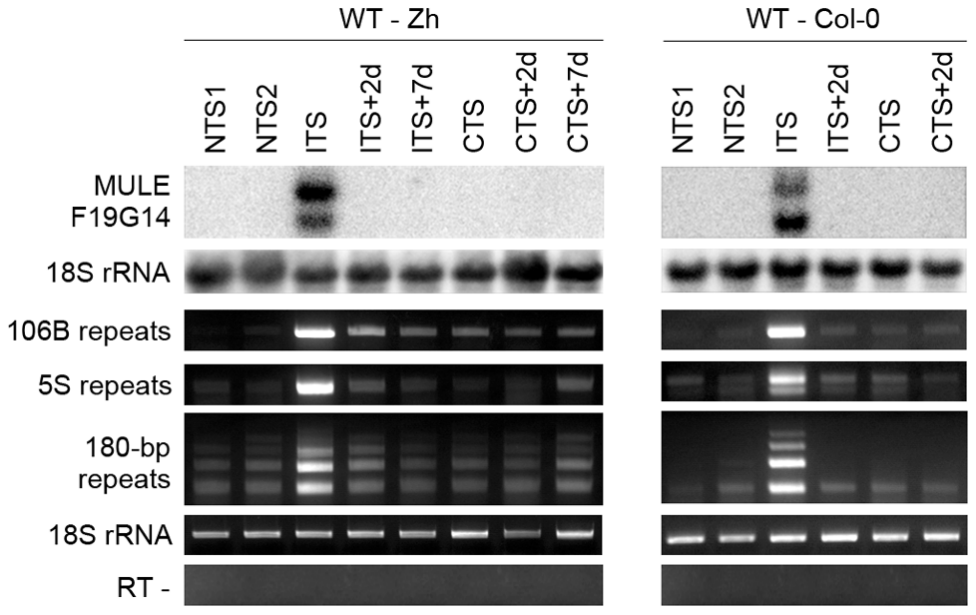
Temperature shift induces transient transcriptional activation of endogenous silent loci. RNA was purified from plants of the Zurich (Zh) and Col-0 accessions after the indicated treatments. Detection of MULE-F19G14 transcripts was performed by Northern blot. Hybridization with an 18S rRNA-specific probe is shown as a loading control. Transcripts corresponding to 106B, 5S and 180-bp repeats were detected by reverse transcription-PCR (RT-PCR). Amplification of 18S rRNA was used to normalize the amounts of RNA template. Negative controls lacked reverse transcriptase (RT -).

To investigate the effect of ITS on silencing of heterochromatic transcription at endogenous targets, we analyzed several silent loci that are activated in mutants impaired in TGS maintenance. First, we determined level of transcripts of a *Mutator*-like transposable element related locus (*MULE-F19G14*, AT2G15810) previously described as strongly transcriptionally activated in the TGS-deficient mutants *mom1* and *ddm1* [54]. We examined the levels of *MULE-F19G14* transcripts in two *Arabidopsis* accessions *Zürich* and *Columbia* (Figure 3). The *MULE-F19G14* remained silent in NTS and CTS in *Zürich* and in *Columbia* plants; however, it was strongly reactivated after ITS in both ecotypes. This suggests that the ITS-induced release of silencing is not restricted to transgenic loci and also not to a particular accession. *MULE-F19G14* RNA was not detected by Northern blot in ITS+2d and ITS+7d plants, indicating that plants of both accessions were equally able to swiftly resilence this endogenous locus.

Although both the transgenic GUS locus and *MULE-F19G14* are silenced by mechanisms contributing to transcriptional suppression in pericentromeric heterochromatin [45], [54], these two targets reside outside of constitutive heterochromatin regions and represent sequences of a single or a few copies. It has been shown recently that a temperature stress of 48 h at 42°C induces transcriptional reactivation of TSI sequences residing in pericentromeric parts of the chromosomes [26]. Similarly, we found that TSI transcripts accumulated in ITS-treated plants (data not shown). To determine whether the ITS would also activate transcription at additional silenced, multicopy sequences incorporated into constitutive heterochromatin, we examined the presence of RNA derived from 180-bp satellite repeats, 106B long terminal-like dispersed repeats and 5S rDNA genes. These repeats are known to be transcriptionally silenced by various epigenetic mechanisms and their transcription is released in mutants impaired in epigenetic regulation of constitutive heterochromatin [27], [29], [45], [48], [54]–[58]. Transcription of all three sets of repeats was induced by ITS but not by CTS (Figure 3) and was also transient, resembling the kinetics observed for the transgenic GUS locus and *MULE-F19G14*. Therefore, we conclude that in both accessions ITS provokes transitory destabilization of silencing of constitutive heterochromatin associated with these various repeats.

### Molecular mechanisms associated with ITS-induced release of transcriptional suppression

**Figure 4:**
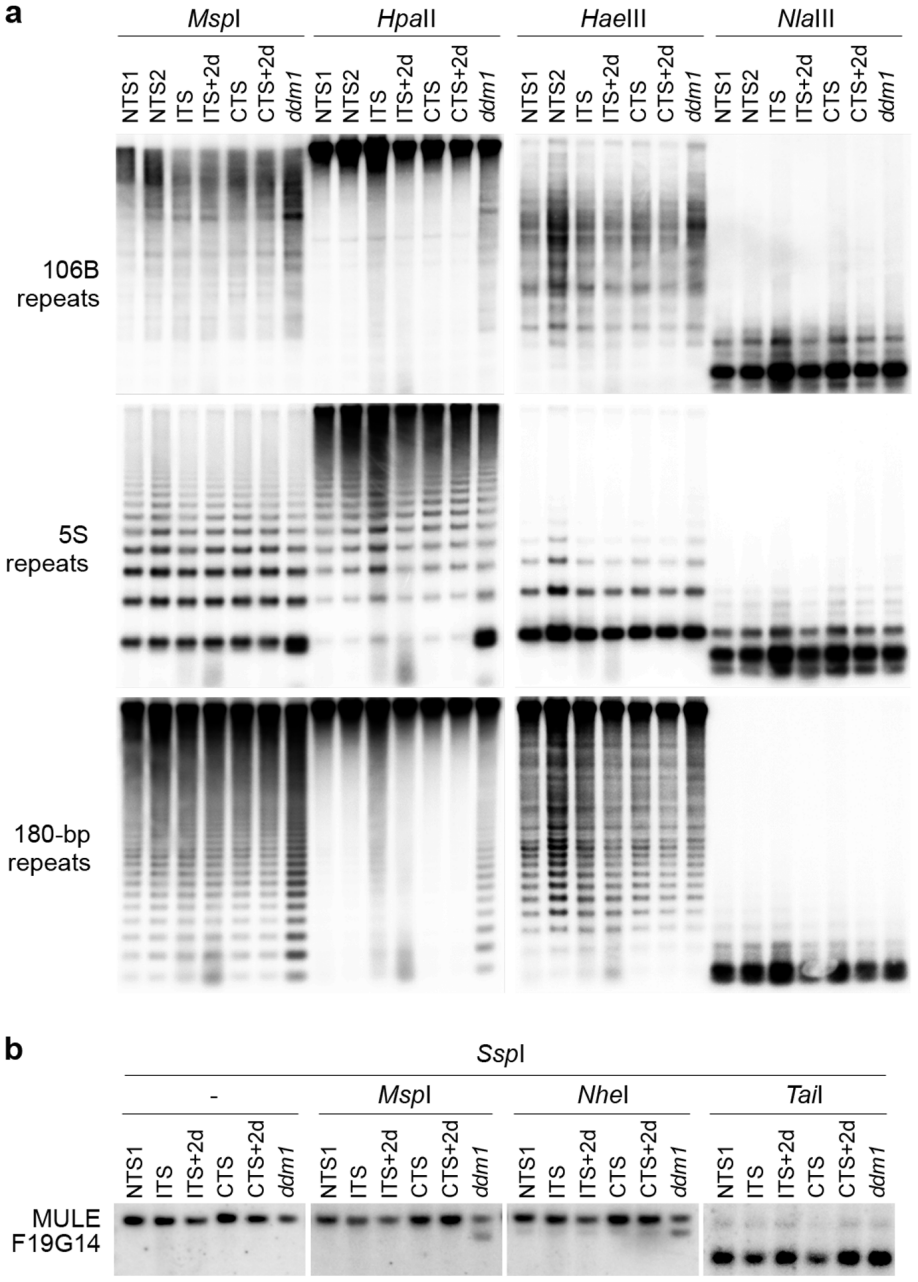
ITS-induced transcriptional activation occurs without detectable changes in the levels of DNA methylation at endogenous loci. (A) Southern blot analysis of DNA methylation at 106B, 5S and 180-bp repeats using the indicated methylation-sensitive restriction endonucleases. (B) Southern blot analysis of DNA methylation at MULE F19G14 was performed by digesting genomic DNAs with SspI (methylation insensitive), followed by digestion with the indicated methylation-sensitive restriction endonucleases.

To determine possible epigenetic mechanisms associated with ITS-induced release of silencing, we first analyzed DNA methylation levels at ITS-sensitive sequences before and after ITS and CTS treatments (Figure 4). Southern blot analyses were performed on genomic DNA digested with *Msp*I (inhibited by methylation of the outer C in the sequence CCGG), *Hpa*II (inhibited by methylation of either C in the sequence CCGG), *Hae*III (inhibited by methylation of the inner C in the sequence GGCC), *Nla*III (inhibited by methylation of the C in the sequence CATG), *Nhe*I (inhibited by methylation of either C in the sequence GCTAGC) and TaiI (reporting on CG methylation). This set of experiments was performed with the *Zürich* ecotype, which withstands ITS conditions better than the *Columbia* ecotype (Figure S1). The DNA methylation-deficient mutant *ddm1-5* available in this ecotype was used as a control. DNA methylation analyses revealed that ITS had no significant influence on methylation levels of cytosines located in either symmetrical (CG or CHG) or asymmetrical (CHH) contexts at the single-copy *MULE-F19G14* (Figure 4B). This is in agreement with a recent finding that a treatment of 48 h at 42°C reactivates transcription of the L5 transgene and of a LINE element without significant changes in DNA methylation [26]. Importantly, DNA methylation status was also maintained at 106B, 5S and 180-bp multicopy targets all residing in constitutive heterochromatin (Figure 4A).

Next, we used chromatin immunoprecipitation (ChIP) to determine the levels of various histone modifications associated with either repressed (histone H3 dimethylation at lysine 9 -H3K9me2, H3K27me2 and H3K27me3) or active transcription (H3K4me3 and H3K9ac-K14ac) at 5S rDNA and 106B repeats and at MULE-F19G14. Compared with CTS and NTS plants, levels of H3K9me2, H3K27me2 and H3K27me3 were unaffected by the ITS, suggesting that activation of transcription following ITS occurred without alteration of the repressive chromatin environment associated with these targets (Figure S2). Levels of H3K4me3 also remained unchanged. However, we detected a slight increase in H3K9ac-K14ac upon ITS similar to previous studies using different stress conditions [26], [59]. Importantly, levels of H3K9ac-K14ac at all examined targets rapidly reverted to the initial level after 2 days of recovery (ITS+2d) (Figure S2).

**Figure 5:**
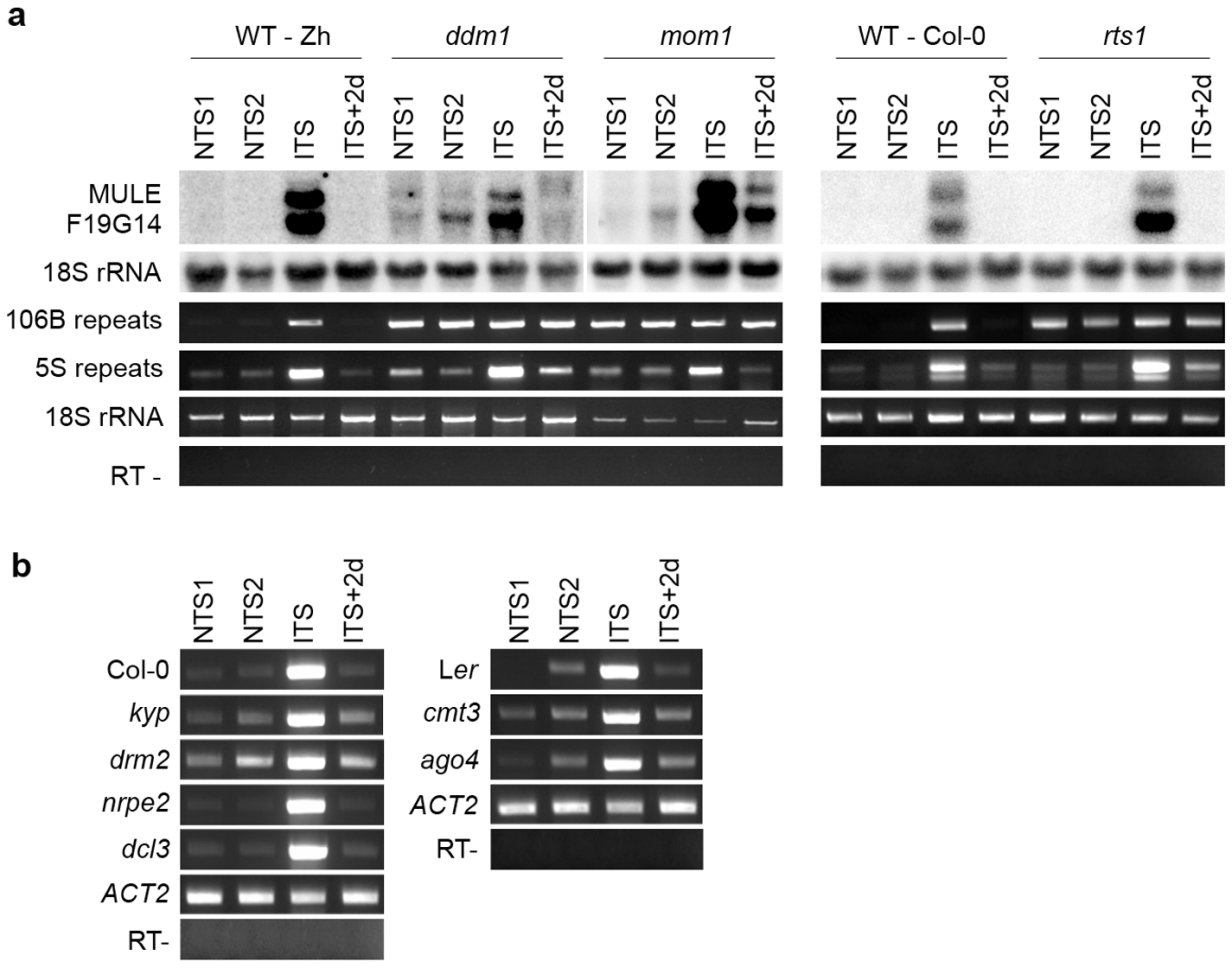
Impact of mutations in epigenetic regulators on ITS-induced transcriptional switches. (A) RNA was extracted from *ddm1*, *mom1* and *rts1* mutant plants and the corresponding wild types (WT) after the indicated treatments. Detection of MULE-F19G14 transcripts was performed by Northern blot. Hybridization with an 18S rRNA-specific probe is shown as a loading control. Transcripts corresponding to 106B, 5S and 180-bp repeats were detected by reverse transcription-PCR (RT-PCR). Amplification of 18S rRNA was used to normalize the amounts of RNA template. Negative controls lacked reverse transcriptase (RT-). (B) RT-PCR analysis of transcripts from 106B repeats in the indicated mutant backgrounds and corresponding WT. Amplification of ACTIN2 (ACT2) RNA was used to normalize the amounts of RNA template. Negative controls lacked reverse transcriptase (RT -).

Since transcriptional activation and swift resilencing of heterochromatin-associated targets following ITS occurred without detectable changes in repressive epigenetic marks (i.e. DNA methylation and histone modifications), we anticipated that factors required for the maintenance of these marks would not be involved in stress-induced transcriptional changes. To test this hypothesis, a *ddm1* mutant was exposed to the ITS conditions. Mutants of the DDM1 SWI2/SNF2 chromatin-remodeling factor show both decreased levels of DNA methylation and alteration of histone H3K9me2 distribution [60], [61]. In agreement with previous reports [29], [54], transcription of MULE-F19G14, 5S rDNA and 106B repeats was induced by the *ddm1* mutation, as revealed by Northern blot and RT-PCR (Figure 5A). The high level of transcripts from 106B repeats in *ddm1* did not significantly increase when mutant plants were stressed, probably because transcriptional reactivation of these sequences had already reached its maximum. However, transcripts originating from MULE-F19G14 and 5S rDNA over-accumulated in *ddm1* plants exposed to ITS compared with *ddm1* NTS plants. Transcript levels returned to the initial state after 2 days (ITS+2d). Together, these results indicate that the transcriptional changes occurring at these targets upon stress are at least in part occurring independently of the DDM1 activity.

The epigenetic regulator MOM1 is required for the maintenance of silencing at loci mostly clustered around centromeric heterochromatin regions [28], [30]. Activation of transcription in *mom1* mutants in these genomic regions takes place with very subtle or no changes in levels and distribution of DNA methylation and histone modifications [28]–[30], [34], [46], [54], [58], resembling release of silencing upon ITS. Therefore, we assessed the possible involvement of MOM1 in stress-induced transcriptional changes. Similar to *ddm1*, 106B repeats transcripts over-accumulated in *mom1* and the transcript level did not further increase when *mom1* plants were subjected to ITS. Transcription of MULE-F19G14 and 5S rDNA repeats was transiently stimulated by ITS in the *mom1* mutant background (Figure 5A), indicating that, like in the DDM1 case, the stress-mediated reactivation of transcription is at least partly independent of MOM1 activity and MOM1 does not participate in subsequent resilencing at these loci.

Our ChIP analysis revealed a modest enrichment in H3K9ac-K14ac at MULE-F19G14, 5S rDNA and 106B repeats following ITS (Figure S2). Previous studies associated the HDA6 histone deacetylase to silencing [45], [47], [62]–[64], and demonstrated that knockdown of this gene, in *rts1-1* mutant, leads to higher levels of H3K9 and H3K14 acetylation [65]. However, Northern blot and RT-PCR assays showed that transcripts from MULE-F19G14 and 5S rDNA over-accumulated in *rts1-1* plants exposed to ITS relative to the NTS control (Figure 5A); after 2 days of recovery, RNA levels of these targets reverted to the non-stressed mutant level. This indicates that HDA6 activity at these targets is not necessary for the transcriptional switches occurring upon stress, resembling DDM1 and MOM1. Similar to *ddm1* and *mom1* mutants, transcripts of 106B repeats over-accumulated in *rts1-1* mutant plants and RT-PCR did not detect further increases when *rts1-1* plants were subjected to ITS.

Next, we examined ITS-triggered transcriptional induction and resilencing in additional mutants deficient in RNA-mediated gene silencing, such as strains deficient in the DRM2 *de novo* DNA methyltransferase, the NRPE2 common subunit of Pol IV and Pol V, the DCL3 endonuclease or the Argonaute protein AGO4. We also tested the impact of mutations in the CMT3 DNA methyltransferase and the KYP/SUVH4 histone H3K9 methyltransferase. All these mutations had no or little effect on transcriptional silencing of 106B repeats (Figure 5B) and showed a stress response similar to their corresponding wild types, in which 106B transcripts accumulated over the levels of the non-stressed plants upon ITS and returned to the initial level in ITS+2d plants (Figure 5B). This shows that none of these silencing effectors are required for either ITS-induced release of transcriptional suppression or the subsequent resilencing.

### Genome-wide analysis of transcriptional changes induced by ITS

**Figure 6:**
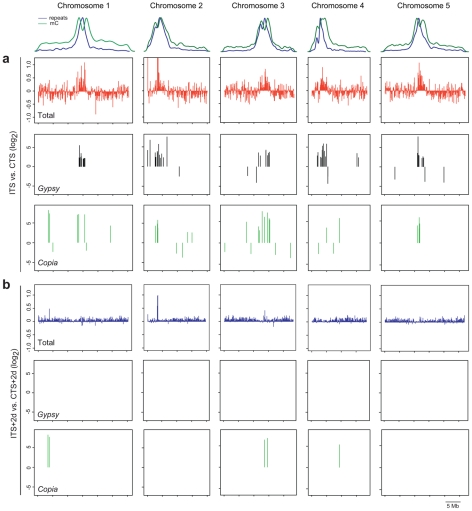
Genome-wide analysis of ITS-induced transcriptional changes. The relative densities of repeats and 5-methylcytosines (mC) along the 5 chromosomes of *Arabidopsis* are shown at the top. (A) Top graphs show chromosome-wide changes in transcript abundance in ITS versus CTS plants in a sliding 100-kb window. Middle and lower graphs represent distribution and variation in transcript accumulation from *gypsy-* and *copia*-type LTR retrotransposons, respectively, in ITS plants compared with CTS plants. (B) Upper graphs represent chromosome-wide changes in transcript accumulation in ITS+2d versus CTS+2d plants in a sliding 100-kb window. Lower graphs indicate distribution and enrichment in *gypsy*- and *copia*-type LTR retroelement transcript in ITS+2d plants compared with CTS+2d plants.

To extend the analysis in an unbiased manner to other ITS-responding chromosomal targets, we determined ITS impact on the whole genome transcriptome with an *Arabidopsis* tiling array. We compared the RNA profiles of wild-type plants exposed to CTS and ITS treatments (Figure 6A). The chromosomal regions with constitutive heterochromatin highly enriched for repeats and DNA methylation, including centromeric, pericentromeric DNA and the heterochromatic knob on chromosome 4, became transcriptionally active following ITS. In contrast, transcription along gene-rich euchromatic parts of the chromosomes was not only stimulated but also often repressed. Overall, we detected differential accumulation of transcripts in ITS plants relative to CTS plants (greater than twofold, *P*<0.01) originating from a total of 6,788 unique annotated genes (TAIR7), with a similar number of genes being either up- (2,890, Table S2) or down-regulated (3,898, Table S3). The number of genes affected by ITS was in the same range as that reported in a previous study using the *Arabidopsis* ATH1 array and plants subjected to various stress conditions [16]. Our tiling array data identified MULE-F19G14 as ITS reactivated and several new targets were further validated using RT-PCR (Figure S3).

As previously described [30], we also included in the analysis TAIR8-annotated transposon sequences. The majority of transposon transcripts over-accumulating upon ITS (greater than fourfold, *P*<0.01, Table S4) correspond to elements residing in constitutive heterochromatin of centromeric and pericentromeric regions, whereas transposons with downregulated transcript levels (less than fourfold, *P*<0.01; Table S5) tend to reside along euchromatic chromosome arms (Figure 6A and Figure S4). Compared with other transposons, in particular transcripts of long terminal repeat (LTR) retrotransposons of the *gypsy* and *copia* groups accumulated after ITS (Figure 6A, Figure S4). Altogether, these results indicate that ITS induces a global release of heterochromatin-associated silencing.

Next, we examined the persistence of ITS-induced transcription on a genome wide scale. For this purpose, we searched among ITS-stimulated transcripts for those that significantly over-accumulated also 2 days post ITS (ITS+2d) in comparison to CTS+2d (greater than twofold for genes and fourfold for transposons, *P*<0.01). In agreement with the analyses of selected targets described above, the vast majority of transcripts originating from either genes or transposons showed no significant difference in their accumulation at ITS+2d compared with CTS+2d plants (Figure 6B), confirming that silencing was globally restored within 2 days of recovery to the initial level prior to ITS. However, there were exceptions to this general rule. For example, a stretch of heterochromatin of chromosome 2 appeared to retain moderate transcriptional activity in ITS+2d plants (Figure 6B). This region corresponds to a probably recent insertion of mitochondrial DNA into the genetically defined centromere of chromosome 2 [66]. In addition, although transcript levels from most transposons diminished, showing no difference in abundance between ITS+2d and CTS+2d plants, some exceptions were detected corresponding to *copia* type LTR retrotransposons with a high level of transcripts persisting 2 days after ITS (Figure 6B, Table S4). Noticeably, these levels were similar to those observed for transcript profiles of plants compared directly after ITS and CTS. This shows that for some transposable elements the kinetics of resilencing after ITS may differ from the general trend.

## Discussion

Early observations on transgenic Petunia plants grown in laboratory conditions or in the field suggested that environmental factors could modulate epigenetic regulation of gene silencing [67]. In general, however, silencing restricting the transcription of sequences within constitutive heterochromatin appears to be highly stable and, so far, its release was observed only in mutants affected in genes encoding epigenetic regulators or in cells subjected to prolonged culture *in vitro* [58], [68]. Although recent studies have reported that transcription of a few pseudogenes, transposons and transposon-derived sequences, in addition to many protein-encoding genes, can be stimulated by abiotic stresses (drought, cold, heat, ABA treatment) [16], [18], [26], here we selected and optimized environmental stress conditions that provoke global release of heterochromatic silencing affecting transcriptional suppression at a high number of targets residing in constitutive heterochromatin.

Following a particular stress treatment involving temperature shifts, alleviation of silencing occurred at many types of sequences residing in pericentromeric and centromeric heterochromatic environment, including tandem-repeat 180-bp satellite sequences, 5S ribosomal DNA arrays, 106B interspersed repeats and transposable elements. The variety of target loci affected by ITS suggests that a particular chromatin context (e.g. association with a specific histone modification/nucleosome density), rather than the primary DNA sequence, determines ITS susceptibility. In this regard, it is of note that stress-induced release of transcriptional silencing is not restricted to loci associated with intermediate heterochromatin (MULE F19G14 and 5S rDNA), which is characteristic of MOM1-regulated targets [28], [29], [54]. This is consistent with the observation that *mom1* mutants can respond to ITS in a similar way to wild-type plants.

It is long known that position effect variegation (PEV) in *Drosophila* can be modulated by ambient temperature changes, with elevated temperatures leading to reduced variegation [69]. In *S. pombe*, silencing of genes located within centromeric regions and of centromeric repeats seems to be temperature sensitive. It has been postulated that this is due to the inhibition of RNA interference (RNAi) that silences transcription at high temperatures [70], [71]. Inhibition of RNAi results in loss of H3K9 methylation associated with heterochromatic silent loci in both *Drosophila* and fission yeast [72], [73]. In *Arabidopsis*, mutations in factors required for RNA-mediated gene silencing also lead to alteration in H3K9 methylation, in addition to a reduction in DNA methylation, showing that DNA and H3K9 methylation are tightly interwoven [60], [74], [75]. We have found that at the examined activated loci the pattern of repressive epigenetic marks typically associated with sequences located in constitutive heterochromatin (dense DNA methylation and H3 methylation at K9 and K27) is not affected by ITS, albeit silencing was efficiently released. Additionally, our analyses revealed that transient induction of transcription still occurs in *ddm1*, *hda6*, *kyp/suvh4*, *cmt3*, *drm2*, *ago4*, *dcl3, nrpe2* and *mom1* mutant plants following ITS treatments. Together, our results strongly suggest that ITS-stimulated transcriptional activity in heterochromatin bypasses the presence of common repressive epigenetic marks and does not depend on known epigenetic regulators. Therefore, ITS possibly counteracts a novel as yet unknown silencing pathway.

A recent study has shown that nucleosomes containing the histone variant H2A.Z are involved in the thermal regulation of transcription [76]. As temperature rises, H2A.Z-carrying nucleosomes are evicted from genes allowing the increase or decrease in their transcriptional activities. Here we observed that the affected chromosomal regions retained high DNA methylation levels despite global alleviation of silencing at heterochromatic sequences following temperature shifts. Given that methylation and the presence of H2A.Z-containing nucleosomes are mutually exclusive [77], we presume that ITS-induced transcriptional activation of heterochromatic sequences most probably occurs independently of H2A.Z deposition/removal.

Plants are sessile, therefore their acclimation to adverse environmental conditions requires swift adaptation by the modulation of gene expression, thereby altering their physiology and ensuring survival. In agreement with previous reports using various stress conditions [16], [18], [78], we have shown that several thousand genes respond at the transcriptional level to the stress we applied. In addition, our particular stress regime released silencing of heterochromatic sequences and transposable elements. In the process of defining optimal stress conditions that destabilize heterochromatin-associated silencing, we observed high lethality when plants were directly shifted from 21°C to 37°C without an intervening period of growth at 4°C. This is reminiscent of the phenomena of cold/heat acclimation required for thermotolerance to extreme temperatures [79]–[82]. Interestingly, we also observed that release of silencing was less efficient when the period at 4°C was omitted. This raises the interesting possibility that efficient release of TGS at specific loci may somehow contribute to thermotolerance.

We observed a drastic impact of ITS on transposon silencing. Although most reactivated sequences were swiftly resilenced 2 days after ITS, elevated transcript levels of some *copia*-type retrotransposons were still detected 48 h after ITS. Previous studies in snapdragon (*Antirrhinum majus*) revealed that a temperature shift induced transposition of the Tam3 DNA transposon [83], [84]. However, transposition was induced by a shift to a lower temperature and was associated with decreased DNA methylation at Tam3, suggesting involvement of a particular epigenetic mechanism in the regulation of Tam3 activity. This seems not to be the case in the transcriptional responses to ITS. For the loci examined in detail that were reactivated by ITS, the transient release of silencing and its re-establishment was independent of changes in DNA methylation levels and classical factors required for small RNA-mediated *de novo* silencing (DRM2, Pol IV/V, DCL3, AGO4). The fact that these factors act through a small RNA guided silencing mechanism also suggests that restoration of silencing following stress occurs independently of small RNA accumulation.

In general, our observations imply that stress-induced destabilization of heterochromatic TGS and its re-establishment use unorthodox and potentially new mechanisms that can now be revealed by forward genetics.

## Materials and Methods

### Plant material and growth conditions

The *mom1-1* [46] and *ddm1-5* [85] strains in the Zurich background, the *ago4-1* [86] and *cmt3-7* [56] strains in the Ler background and the *drm2-2* [87], *kyp-7* [32], *nrpd2a-2/nrpe2* [88], *dcl3-1* [75] and *rts1-1* [62] strains in the Col-0 background have been described previously. The transgenic L5 line [44] was kindly provided by H. Vaucheret.

Seeds were surface sterilized in 0.4% sodium-hypochlorite/80% ethanol for 10 min, washed 3 times with 100% ethanol and dried under a laminar-flow hood. Seeds were then plated on Murashige and Skoog (½ MS) medium (Duchefa) containing 0.6% agar and stratified at 6°C for 2 days. Plates were incubated in growth chambers at 21°C with 12 h light/ 12h dark. For cold treatment, plates were incubated at 4°C for the indicated times with the same photoperiod. For the ITS treatment, plates were incubated in a growth chamber at 37°C for 15 h (12 h light/3 h dark).

### Histochemical GUS staining

Staining was performed on whole-seedlings with 5-bromo-4-chloro-3-indolyl-b-glucuronic acid, 0.5 mM potassium ferricyanide, 0.5 mM potassium ferrocyanide, 10 mM EDTA and 50 mM sodium phosphate buffer pH 7.2 [53].

### Chromatin immunoprecipitation

ChIP was performed as described previously [57]. The histone-DNA complexes were immunoprecipitated with α-dimethyl H3K9 (abcam, ab1220), α-dimethyl H3K27 (Millipore, 07-322), α-trimethyl-H3K27 (a kind gift from T. Jenuwein), α-trimethyl-H3K4 (Millipore, 07-473), or α-acetyl-H3K9-K14 (Millipore, 06-599). Subsequent PCR reactions were performed in 20 µl final volume, starting with 5 min at 95°C and followed by 18–34 cycles (depending on the region being amplified) of 95°C, 60°C (54°C for 5S rDNA; 55°C for 106B repeats), and 72°C (30 s each) with a final elongation of 5 min at 72°C. PCR products were scanned with a Molecular Imager FX (Bio-Rad) after electrophoretic separation and quantified using the Quantity One software (Bio-Rad). Primers are described in Table S1.

### Southern blot, Northern blot, and Reverse Transcription PCR

Southern blot analyses were performed as described previously [29]. Total RNA was extracted from whole seedlings using TRI reagent (Sigma). For Northern blots, 10 µg of total RNA per lane was used. Probes were labeled with [α-^32^P]dCTP using random hexamer priming (Megaprime DNA labeling system, GE Healthcare). RT-PCR analyses were performed as described previously [29]. Primers are listed in Table S1.

### Transcription profiling

Plants of the Zürich accession were grown under conditions described above and RNA was extracted from whole seedlings using the Ambion mirVana miRNA isolation kit as described previously [89]. Subsequent steps were performed as described previously, using the GeneChip *Arabidopsis* Tiling 1.0R array from Affymetrix [30]. Chip data have been submitted to Gene Expression Omnibus (GEO, GSE23243) and can be visualized using the EpiExpress browser at http://gbrowse.vital-it.ch/cgi-bin/gbrowse/epiexpress/.

### Note added in proof

Parts of this work are consistent with data described in a parallel publication (Pecinka A., Dinh H. Q., Baubec T., Rosa M., Lettner N., and Mittelsten Scheid O. [2010] Epigenetic regulation of repetitive elements is attenuated by prolonged heat stress in *Arabidopsis.* Plant Cell, online).

## Supporting Information

Figure S1 Plants of the Zürich ecotype display a better fitness following ITS than plants of the Columbia ecotype. (a) Wild-type seedlings of the Columbia (WT-Col-0, left) and the Zürich ecotypes (WT-Zh, right) grown *in vitro* under the indicated conditions. (b) Enlargement of plates shown in (a). Unlike WT-Zh, some WT-Col-0 seedlings did not survive the ITS treatment (white seedlings; compare WT-Col-0 ITS+2d and WT-Zh ITS+2d).
(0.26 MB PDF)

Figure S2 Impact of ITS on histone post-translational modifications. (a) Input and mock controls of ChIP analysis of *MULE F19G14, 106B* repeats and *5S* repeats using antibodies specific for (b) H3K4me3 and H3K9ac-K14ac , which are associated with active transcription, and for (c) H3K9me2, H3K27me3 and H3K27me2, which are associated with repressed transcription. Representative gels are shown. The *TUBULIN8 (TUB8)* was used to normalize the amount of DNA. *MULE F19G14, 106B* repeats and *5S* repeats reproducibly show a slight enrichment in H3K9ac-K14ac upon ITS (b). (d) The *met1-3* mutant (Col-0 genetic background) was used as a control for the ChIP procedure and showed expected enrichment in H3K4me3 and concomitant decrease in H3K9me2 at *106B* and *5S* repeats relative to wild-type (WT) plants.
(0.30 MB PDF)

Figure S3 Tiling Array data and RT-PCR validation. (a) Relative accumulation transcripts from selected loci (*MULE F19G14, ROS1, DML2* and *SDC*) comparing ITS and CTS from the tiling array data. (b) RT-PCR validation of the tiling data of the slightly differentially expressed targets, *ROS1, DML2* and *SDC*, after ITS compared with CTS; amplification of 18S rRNA was used to normalize the amounts of RNA template, and the negative control lacked reverse transcriptase (RT -).
(0.17 MB PDF)

Figure S4 Genome-wide analysis of ITS-induced transcriptional changes at transposons. The upper plots show the relative densities of repeats (blue lines) and DNA methylation (green lines) along the 5 chromosomes of *Arabidopsis*. Graphs show the chromosome-wide distribution and variation in transcript abundance (Log2 scale) of transposons (grouped by superfamilies) after ITS versus CTS.
(0.32 MB PDF)

Table S1 List of primers used for RT-PCR and/or chromatin immunoprecipitation analysis.
(0.03 MB DOC)

Table S2 List of genes significantly upregulated (greater than twofold, P<0.01) in ITS versus CTS. The comparison of transcript levels between ITS+2d and CTS+2d for the corresponding genes is also presented.
(0.38 MB XLS)

Table S3 List of genes significantly downregulated (greater than twofold, P<0.01) in ITS versus CTS. The comparison of transcript levels between ITS+2d and CTS+2d for the corresponding genes is also presented.
(0.50 MB XLS)

Table S4 List of transposable elements significantly upregulated (greater than fourfold, P<0.01) in ITS versus CTS. Transposons for which transcripts still over-accumulate in ITS+2d compared with CTS+2d, are listed at the end of the Table.
(0.11 MB XLS)

Table S5 List of transposable elements significantly downregulated (greater than fourfold, P<0.01) in ITS versus CTS.
(0.04 MB XLS)
