# Supplementary figures and images for: Stress-Induced Activation of Heterochromatic Transcription
Source: PLoS Genet. 2010 Oct 28;6(10):e1001175. doi: 10.1371/journal.pgen.1001175 (PMC2965753; doi:10.1371/journal.pgen.1001175)

**a**

WT - Col-0

WT - Zh

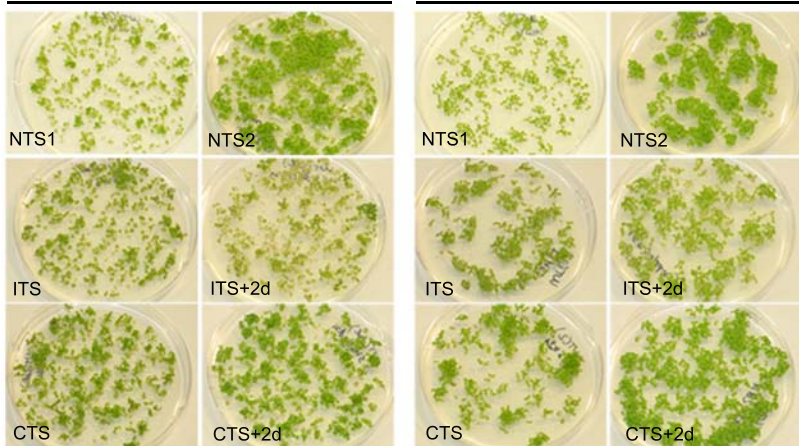**b**

WT - Col-0

WT - Zh

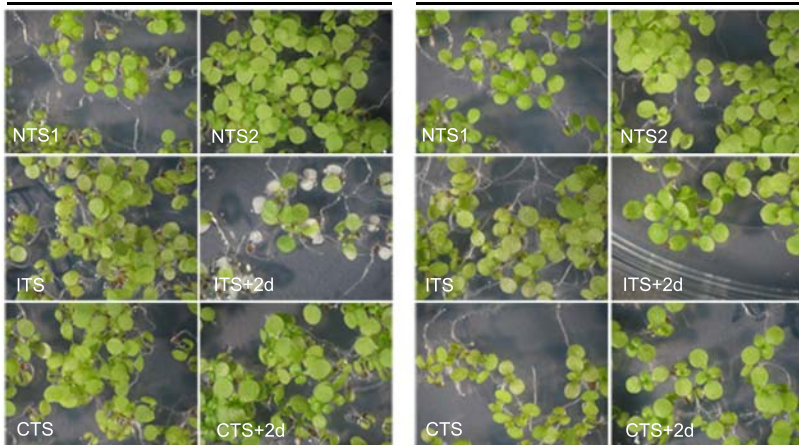

Figure S1

Supplement: Figure S1 — Plants of the Zürich ecotype display a better fitness following ITS than plants of the Columbia ecotype. (a) Wild-type seedlings of the Columbia (WT-Col-0, left) and the Zürich ecotypes (WT-Zh, right) grown in vitro under the indicated conditions. (b) Enlargement of plates shown in (a). Unlike WT-Zh, some WT-Col-0 seedlings did not survive the ITS treatment (white seedlings; compare WT-Col-0 ITS+2d and WT-Zh ITS+2d). (0.26 MB PDF) [file pgen.1001175.s001.pdf]

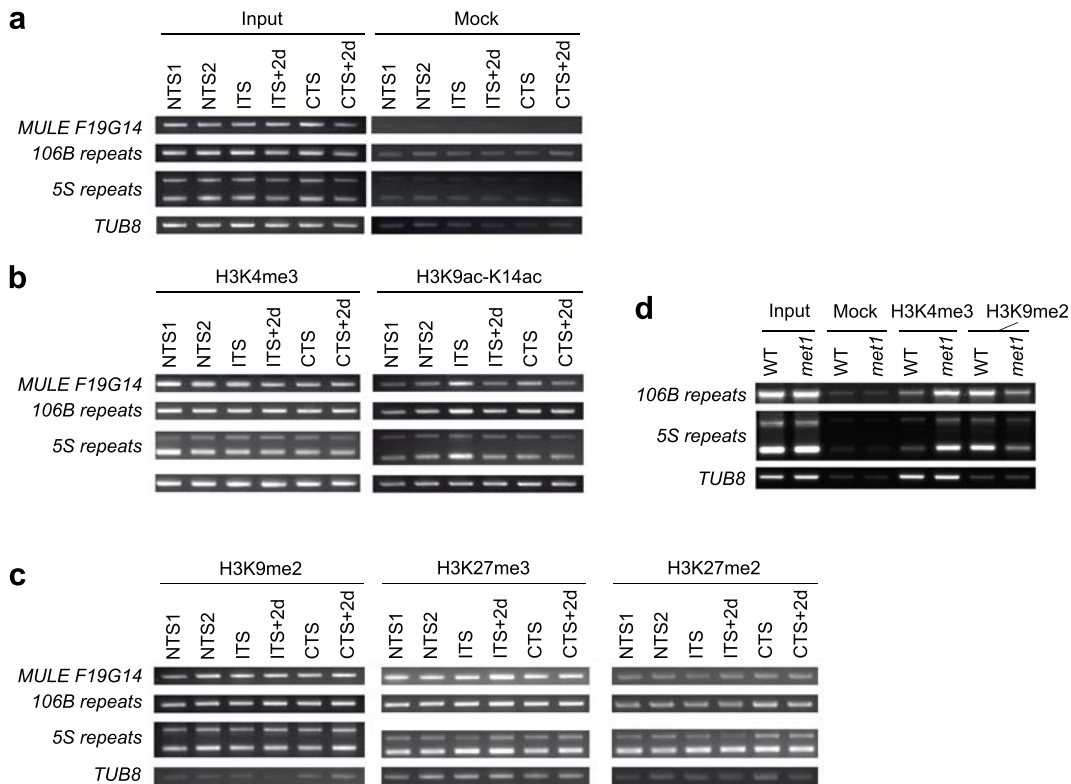

Supplementary Figure 2

Supplement: Figure S2 — Impact of ITS on histone post-translational modifications. (a) Input and mock controls of ChIP analysis of MULE F19G14, 106B repeats and 5S repeats using antibodies specific for (b) H3K4me3 and H3K9ac-K14ac , which are associated with active transcription, and for (c) H3K9me2, H3K27me3 and H3K27me2, which are associated with repressed transcription. Representative gels are shown. The TUBULIN8 (TUB8) was used to normalize the amount of DNA. MULE F19G14, 106B repeats and 5S repeats reproducibly show a slight enrichment in H3K9ac-K14ac upon ITS (b). (d) The met1-3 mutant (Col-0 genetic background) was used as a control for the ChIP procedure and showed expected enrichment in H3K4me3 and concomitant decrease in H3K9me2 at 106B and 5S repeats relative to wild-type (WT) plants. (0.30 MB PDF) [file pgen.1001175.s002.pdf]

**a**

| Locus                               | ITS vs. CTS ( $\log_2$ ) |
|-------------------------------------|--------------------------|
| AT2G15810<br>( <i>MULE-F19G14</i> ) | 5.6                      |
| AT2G36490<br>( <i>ROS1</i> )        | -1.6                     |
| AT3G10010<br>( <i>DML2</i> )        | -1.4                     |
| AT2G17690<br>( <i>SDC</i> )         | 1.2                      |

**b**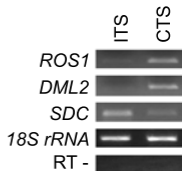

Figure S3

Supplement: Figure S3 — Tiling Array data and RT-PCR validation. (a) Relative accumulation transcripts from selected loci (MULE F19G14, ROS1, DML2 and SDC) comparing ITS and CTS from the tiling array data. (b) RT-PCR validation of the tiling data of the slightly differentially expressed targets, ROS1, DML2 and SDC, after ITS compared with CTS; amplification of 18S rRNA was used to normalize the amounts of RNA template, and the negative control lacked reverse transcriptase (RT -). (0.17 MB PDF) [file pgen.1001175.s003.pdf]

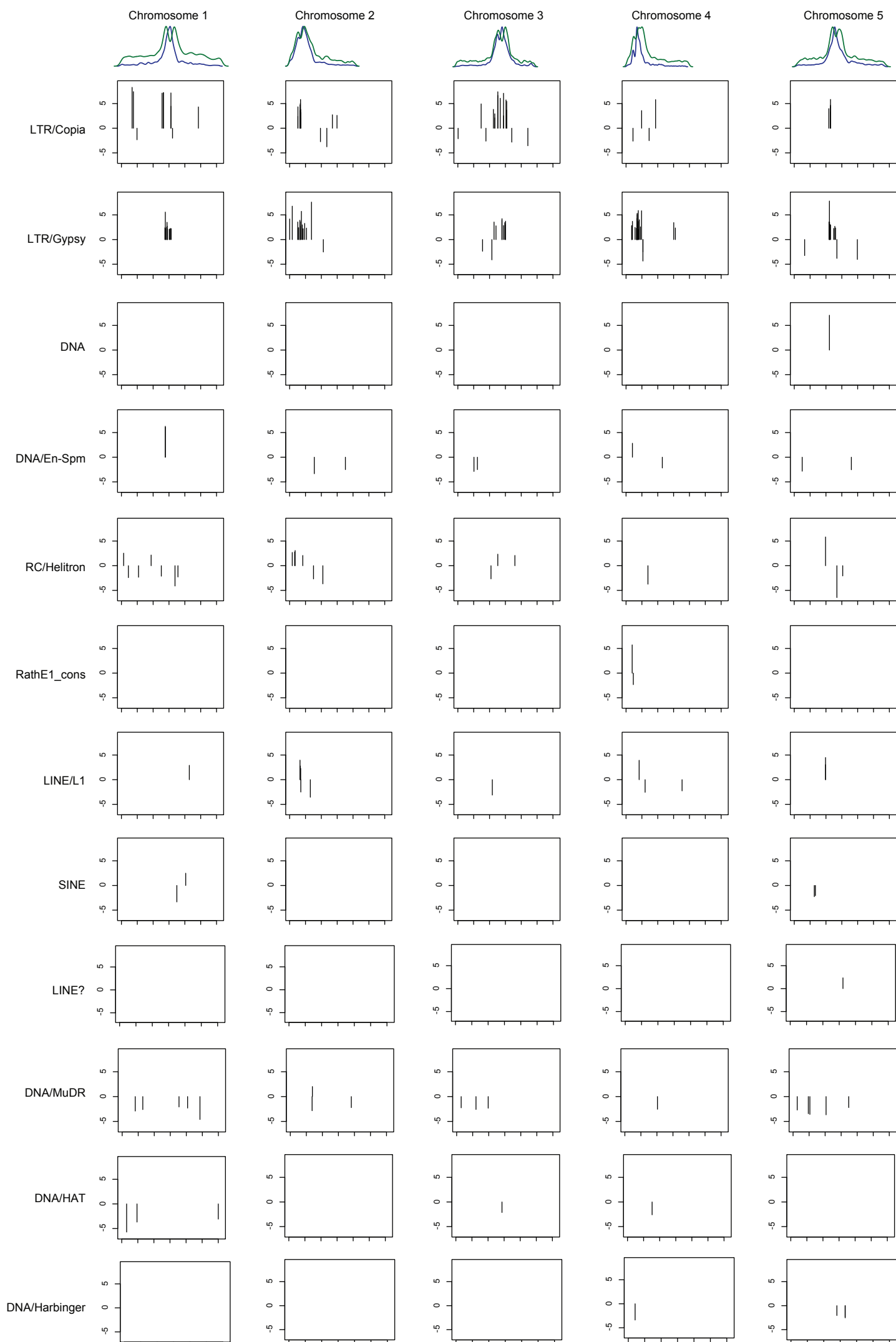

Figure S4

Supplement: Figure S4 — Genome-wide analysis of ITS-induced transcriptional changes at transposons. The upper plots show the relative densities of repeats (blue lines) and DNA methylation (green lines) along the 5 chromosomes of Arabidopsis. Graphs show the chromosome-wide distribution and variation in transcript abundance (Log2 scale) of transposons (grouped by superfamilies) after ITS versus CTS. (0.32 MB PDF) [file pgen.1001175.s004.pdf]
