# Supplementary material for: Stress-Induced Activation of Heterochromatic Transcription
Source: PLoS Genet. 2010 Oct 28;6(10):e1001175. doi: 10.1371/journal.pgen.1001175 (PMC2965753; doi:10.1371/journal.pgen.1001175)
Supplement: Table S1 — List of primers used for RT-PCR and/or chromatin immunoprecipitation analysis. (0.03 MB DOC) [file pgen.1001175.s005.doc]

**Table S1.**

**Target Primer Sequence**

*106B* repeats 106B-F TTGATTGATAGATCCCTTCTGGA

106B-R CGAGGATGGGGTAATTGAGT

*18S rRNA* 18S-F TGGTTGATCCTGCCAGTAGTCA

18-R CACGTAGCTAGTTAGCAGGCTG

*180-bp* repeats 180(all)-F ACCATCAAAGCCTTGAGAAGCA

180(all)-R CCGTATGAGTCTTTGTCTTTGTATCTTCT

*5S repeats* RTPCR5S1 GGATGCGATCATACCAG

5SUNIV2 CGAAAAGGTATCACATGCC

*Actin2* Act2-F CTAAGCTCTCAAGATCAAAGGC

Act2-R AACATTGCAAAGAGTTTCAAGG

*DML2* At3g10010-F GACGCCAGGTGAAACCGTGA

At3g10010-R GAACATCCATCCATTGCCCA

*GUS* GUS-F1 CGTTGGTGGGAAAGCGCGTT

GUS-R1 TCACCATTCCCGGCGGGATA

*MULE F19G14* 1904 CGTCCATGCTTATCCTTATCCCAT

1915 CTAATCGTGTTCTACTTCACACGAC

*ROS1*  ROS1-F AGAAGAAATTCCTACCATCA

ROS1-R ACCGTTCTTCGAGGTAATTC

*SDC* SDC-RT-F TCTCCGGACCAAGGGTGGTT

SDC-RT-R CCTGATGATGTCGTAAGCGGAACT

*Tubulin8* TUB8-F ATAACCGTTTCAAATTCTCTCTCTC

TUB8-R TGCAAATCGTTCTCTCCTTG
